# The effect of sampling height on grass pollen concentrations in different urban environments in the Helsinki Metropolitan Area, Finland

**DOI:** 10.1371/journal.pone.0239726

**Published:** 2020-09-29

**Authors:** Timo T. Hugg, Mirkka Tuokila, Sanna Korkonen, Jan Weckström, Maritta S. Jaakkola, Jouni J. K. Jaakkola

**Affiliations:** 1 Center for Environmental and Respiratory Health Research, University of Oulu, Oulu, Finland; 2 Medical Research Center Oulu, Oulu University Hospital and University of Oulu, Oulu, Finland; 3 Environmental Change Research Unit (ECRU), Ecosystems and Environment Research Programme, Faculty of Biological and Environmental Sciences and Helsinki Institute of Sustainability Science (HELSUS), University of Helsinki, Helsinki, Finland; George Mason University, UNITED STATES

## Abstract

**Introduction:**

It is important to study potential differences in pollen concentrations between sampling heights because of diverse outdoor and indoor activity of humans (exposure) at different height levels in urban environments. Previous studies have investigated the effect of height on pollen concentrations based on just one or a few sampling points. We studied the effect of sampling height on grass pollen concentrations in several urban environments with different levels of urbanity.

**Methods:**

This study was conducted in the Helsinki Metropolitan Area, Finland, in 2013 during the pollen season of grasses. Pollen grains were monitored in eight different points in the morning and afternoon. Rotorod-type samplers were attached on sampling poles at the heights of 1.5 meters and 4 meters.

**Results:**

Grass pollen concentrations were on average higher at the height of 1.5 meters (Helsinki mean 5.24 grains / m^3^; Espoo mean 75.71 grains / m^3^) compared to the height of 4 meters (Helsinki mean 3.84 grains / m^3^; Espoo mean 37.42 grains / m^3^) with a difference of 1.40 grains / m^3^ (95% CI -0.21 to 3.01) in Helsinki, and 38.29 grains / m^3^ (7.52 to 69.07) in Espoo, although not always statistically significant. This was detected both in the morning and in the afternoon. However, in the most urban sites the levels were lower at 1.5 meters compared to 4 meters, whereas in the least urban sites the concentrations were higher at 1.5 meters. In linear regression models with interaction terms, the modifying effect of urbanity on concentration-height relation was statistically significant in both cities. The effect of urbanity on pollen concentrations at both heights was stronger in less urban Espoo.

**Conclusions:**

The present study provides evidence that height affects the abundance and distribution of grass pollen in urban environments, but this effect depends on the level of urbanity.

## 1 Introduction

Pollen allergy forms a major health problem globally. Due to global occurrence, grass pollen has been ranked as the most important aeroallergen and the main cause of pollen-related allergy worldwide [1, 2]. Theoretically, subjects suffering from pollen allergy can try to reduce their exposure to pollen grains by spending more time indoors or outdoors in environments where pollen concentration are known to be low. Such environments include indoor spaces with efficient filter system [3] and outdoor spaces that are surrounded by a limited number of vegetation, e.g. the most urban environments [4].

In previous studies, the number of grass pollen grains has been shown to decrease with height [5–7], or increase with height [8, 9], or has not change with height [10, 11], or has been shown a varying trend with increasing height [12]. Thus, the results have so far been contradictory with each other. Recently, Rojo et al. [13] have estimated the near-ground vertical profile of pollen concentrations based on data from 25 areas and 59 pollen stations. They found that pollen concentrations decreased with height and daily fluctuations in pollen concentrations were stronger at near-ground level (< 10 m above ground) compared to higher levels. Other factors that have been suggested to affect the distribution and dispersion of pollen grains include the location and height of flowers, the period of pollination, the number and characteristics of pollen grains released, and the habitat and meteorological conditions.

According to Romero-Morte et al. [14], a small number of species were responsible for majority of the airborne grass pollen and critical factors affecting the contribution were flowering phenology, pollen size, abundance of the species and pollen production in Toledo, Spain. It has been suggested that the pollen dispersal capacity of several species of grasses is limited, because the size of pollen grain is relatively large and because pollen grains are released close to the ground [15]. However, grass pollen concentrations can periodically reach moderate or even high levels, even in environments where there is no vegetation nearby [4].

Only a little is known about the distribution and abundance of pollen grains at different heights in different urban environments. In previous studies, pollen concentrations were mostly monitored only in one site, or they were compared between the breathing height, i.e. 1.5 meters above the ground, and the roof level, i.e. 15–20 meters above the ground [5, 6, 10, 11, 16]. No previous study has measured urban pollen concentrations at two different heights in several different sampling sites following urban-rural gradient.

In the present study, we monitored grass pollen concentrations at the heights of 1.5 and 4 meters in eight urban environments in the Helsinki Metropolitan Area, Finland. The objectives of this study were 1) to investigate how pollen grains of grasses are distributed across urban space between two heights during the pollen season of grasses and, 2) to assess the potential role of different determinants in affecting the distribution and abundance of grass pollen at two heights. This is the first study that investigates such relations extensively and we were able to provide evidence that height affects the abundance and distribution of grass pollen in urban environments, but this effect depends on the level of urbanity.

## 2 Materials and methods

### 2.1 Study areas

This study was conducted in the neighboring cities of Helsinki (60°10′15″N, 024°56′15″E) and Espoo (60°12′20″N, 024°39′20″E) in Southern Finland. Helsinki is the capital and the largest city of Finland with 622 000 inhabitants. Espoo is the second largest city in Finland, with 266 000 inhabitants. Helsinki, Espoo and the surrounding cities comprise the Helsinki Metropolitan area with altogether 1.1 million inhabitants. The population density is 2.930 and 858 inhabitants per km^2^ in Helsinki and in Espoo, respectively (*Statistics; www.HelsinkiRegion.fi*). The study area is located in Southern Finland between the Baltic Sea and the Eurasian continent, and thus, has characteristics of both maritime and continental climates [4]. The mean annual air temperature and the mean monthly July air temperature are +5.9°C and +17.8°C, respectively. The mean annual precipitation and the mean monthly July precipitation are 655 mm and 63 mm, respectively, in these study areas (*Finnish Meteorological Institute;* *http*:*//ilmatieteenlaitos*.*fi/*). Grasses constitute a typical plant group in the ground layer of the area. The two study areas typically include both the effectively constructed urban environments with limited amount of vegetation and more or less natural environments covered by diverse vegetation.

### 2.2 Assessment of urbanity

Urbanity of each sampling site was evaluated using two approaches. First, a city-specific urban gradient ranking from 1 (most urban) to 4 (least urban) was conducted visually applying the following criteria: construction efficiency (scoring 1–10), street network coverage (1–10), and quantity of vegetation (10–1). Zero to ten points were given for each category of assessment (1 = natural environment and/or extremely lightly modified environment, 10 = man-made and/or extremely strongly modified environment). As an exception to such scoring, for quantity of vegetation 1 referred to man-made and 10 to natural environments). The division of each category followed so called 10% rule. Each change in urban gradient score corresponded to about 10% change in construction efficiency, street network coverage and/or quantity of vegetation. The scoring of each sampling site was based on total points given by two researchers reflecting the opinion of both of them [4]. Secondly, urbanity was assessed by applying standardized Separated Land Use and Land Cover Information System [17] (Table 1). The surface area of urban land use was estimated in the sampling sites. Raynor et al. [18] have shown that the concentrations of Timothy grass pollen decrease rapidly in relation to distance from the pollen source. The pollen concentrations at the distance of 80 meters from the edge of pollen source were typically only 5% from the concentrations found in the edge of pollen source. Because of limited dispersal capacity of grass pollen, urban land use within a 100-meter radius from the sampling sites were used in the analyses.

**Table 1:** Characteristics of the study sites along the urban-rural gradient in the cities of Helsinki and Espoo.

Study site	Construction Efficiency^c^	Street Network Coverage^d^	Quantity of Vegetation^e^	Urban Land Use (area per 100m radius)^f^
**Helsinki 1**^**a**^	9	7	1	30 100
**Helsinki 2**	8	5	2	28 700
**Helsinki 3**	3	4	7	24 800
**Helsinki 4**^**b**^	4	4	8	21 800
**Espoo 1**^**a**^	7	5	4	21 200
**Espoo 2**	5	3	5	28 800
**Espoo 3**	3	3	7	14 500
**Espoo 4**^**b**^	1	1	9	3 700

^a^Most urban.

^b^Least urban.

^c^Points of construction efficiency (1–10), where 1 represents natural environment and/or extremely lightly modified environment and correspondingly 10 represents man-made and/or extremely strongly modified environment.

^d^Street network coverage (1–10), where 1 represents natural environment and/or extremely lightly modified environment and correspondingly 10 represents man-made and/or extremely strongly modified environment.

^e^Quantity of vegetation (10–1), where 10 represents natural environment and/or extremely lightly modified environment and correspondingly 1 represents man-made and/or extremely strongly modified environment.

^f^Surface area of urban land use in square meters within a 100-meter radius of the study site. The highest values reflect the highest level of urbanization.

### 2.3 Assessment of grass pollen levels

**Fig 1:**
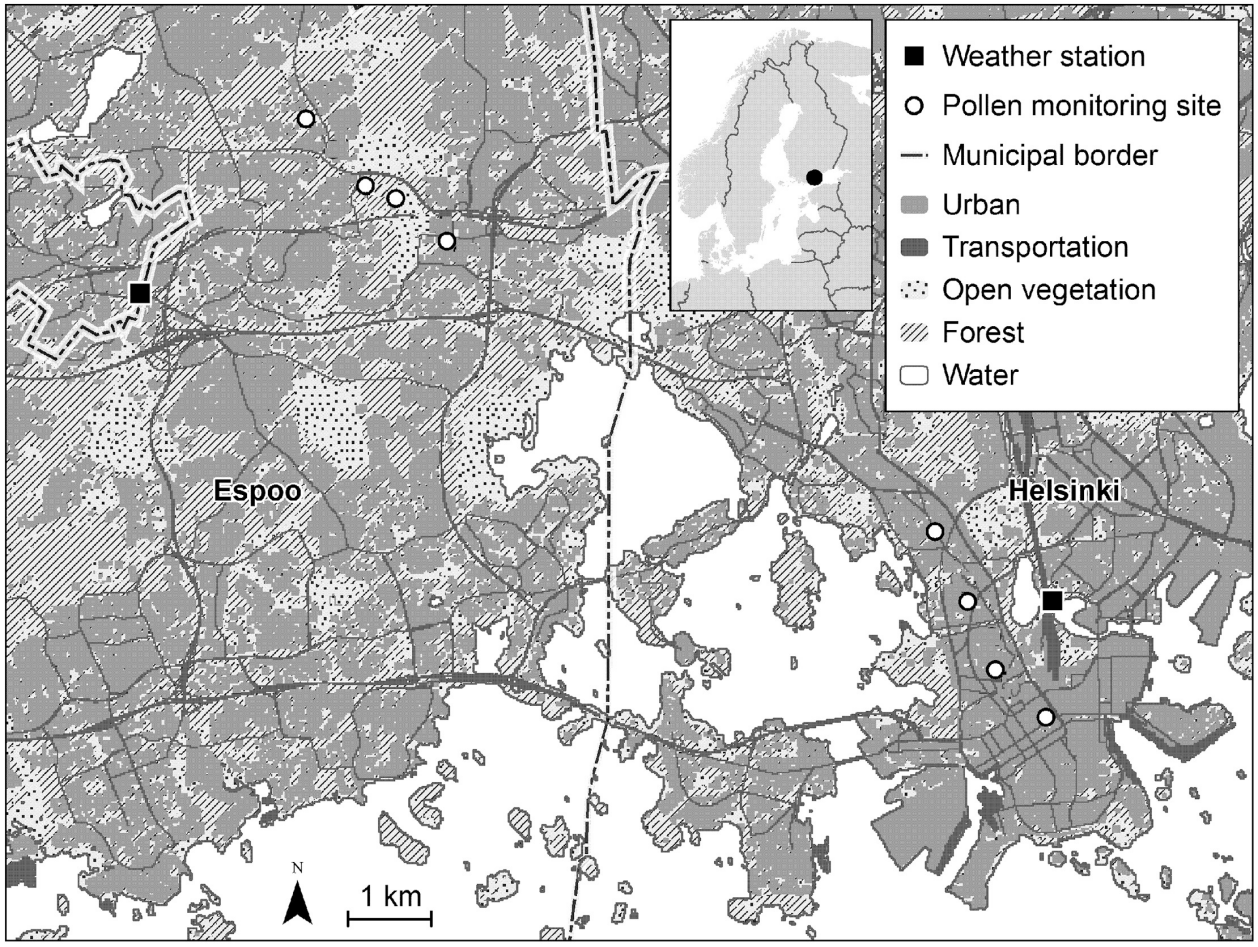
Map of the study area. Sampling sites are presented as open circles (modified from the previous publication [4]). Background map contains data from the General map 1:4 500 000 by the National Land Survey of Finland and the CLC2012 dataset by SYKE (partly Metla, Mavi, LIVI, VRK, MML Maastotietokanta 05/2012), licensed under a Creative Commons BY 4.0 International License (http://creativecommons.org/licenses/by/4.0/).

Pollen sampling was conducted at two lines of three kilometers that captured the urban-rural gradient within the cities of Helsinki and Espoo [4; Fig 1]. Permissions for the sampling were asked and received from the housing cooperatives. Grass pollen concentrations (exposure) were monitored simultaneously at the breathing height, i.e. 1.5 meters, and at the height of 4 meters at 8 locations using rotorod-type samplers [19]. Samplers were equipped with a U-shaped metal rod (1.7 mm in diameter) and a power source (battery: NX, Powerfit S312/1.2S, Part No: AMP9033). Battery was fully charged at the start of each day. Transparent Melinex tape coated with an adhesive (Vaseline) was fixed to the upper ends of the measurement rods. The speed of rotation of each sampler was monitored with a Shimpo Instruments’ (Itasca, Illinois, US) Shimpo DT-201 digital tachometer to ensure correct performance. The average speed of rotation of the arms was 2,173 rpm, varying between 2,040 and 2,275 rpm.

Samplers were attached to the top of the sampling poles. Both samplers operated at the same time. The sampling was conducted on days with no rainfall during the grass pollen season, i.e. between 27 June and 21 July 2013. The number of sampling days was 24 and the total number of observations was 293 in Helsinki and 311 in Espoo. The sampling period was 30 minutes and started on the hour. Sampling was conducted at each site twice a day: in the morning between 8.00 and 11.30 am and in the afternoon between 1 pm and 4.30 pm. The sampling progressed in time and was conducted within lines as a cycle of four days (S1 Table) [4]. The protocol was designed to provide a balanced assessment of spatio-temporal variation in pollen concentrations.

Pollen concentrations were determined by optical microscopic (Olympus BX43, Olympus Corporation, Tokyo, Japan) counting from the collection tapes with 400 x magnification. A single transect line in the middle of the sample was examined; this comprised 32% of the total sample [20–22]. Pollen measurements were converted into volumetric equivalents, expressed as the amount of pollen grains per cubic meter of air sampled (grains/m^3^) [23].

### 2.4 Covariates

Urban gradient (sampling sites 1–4), urban land use (area per 100 m radius), measurement period (i.e. morning or afternoon), day (i.e. weekday or weekend) and temperature were investigated and adjusted as covariates in the multivariate analyses. Weather-related data were obtained from the Finnish Meteorological Institute. The two background weather stations were located in Kaisaniemi, Helsinki, and in Sepänkylä, Espoo. The distance from the background weather stations to the sampling sites varied from 1.1 to 2.3 km in Helsinki and from 2.9 to 3.8 km in Espoo.

### 2.5 Statistical methods

The graphical illustration of data and the Kolmogorov-Smirnov Goodness-of-Fit test indicated that the data were not normally distributed. Therefore, we applied non-parametric statistical methods. We used the Wilcoxon two-sample (Two-Sided probability) test for paired comparison of daily pollen concentrations to analyze potential differences between the lower and upper sampling sites. Because of the zero-inflated distribution of pollen data, negative binomial regression modelling (applying proc GENMOD procedure) was used to assess the relation between environmental factors (as independent variables) and pollen concentration (as dependent variable) at the two heights. In the elaborative analyses we observed that the height-concentration relation varied according to the level of urbanity. We assessed this formally in the regression models by fitting an interaction term height*urban gradient or height*urban land use. The results of two rods were analyzed as two separate measurements. Analyses were performed applying the SAS software (*SAS 9*.*4*, *SAS Institute*, *Inc*., *Cary*, *NC*, *U*.*S*.). Arithmetic means, medians, and minimum and maximum values of the grass pollen are presented in Table 2.

**Table 2:** Mean, median, minimum and maximum values of grass pollen concentrations (pollen grains/m^3^) in relation to height and study period at the study sites in the cities of Helsinki (n = 293) and Espoo (n = 311).

	Morning, height 1.5m (n) / 4.0m (n)			Afternoon, height 1.5m (n) / 4.0m (n)		
Study site	Mean	Median	Min-Max	Mean	Median	Min-Max
**Helsinki 1**^**a**^	2.55 (22) / 3.36 (22)	1.5 / 3.0	0–17 / 0–11	4.38 (16) / 4.00 (16)	3.0 / 3.0	0–23 / 0–14
**Helsinki 2**	1.29 (24) / 2.13 (24)	0.0 / 0.0	0–9 / 0–11	2.94 (16) / 2.94 (16)	3.0 / 3.0	0–11 / 0–11
**Helsinki 3**	2.76 (21) / 3.68 (22)	3.0 / 3.0	0–9 / 0–23	4.47 (15) / 3.93 (14)	6.0 / 3.0	0–14 / 0–11
**Helsinki 4**^**b**^	15.79 (19) / 5.53 (17)	3.0 / 3.0	0–119 / 0–20	9.50 (16) / 6.69 (13)	6.0 / 6.0	0–45 / 3–20
**Espoo 1**^**a**^	3.59 (22) / 3.77 (22)	1.5 / 3.0	0–20 / 0–11	5.40 (20) / 5.15 (20)	6.0 / 3.0	0–11 / 0–23
**Espoo 2**	3.04 (24) / 4.13 (24)	3.0 / 3.0	0–14 / 0–14	7.20 (20) / 6.90 (20)	3.0 / 4.5	0–34 / 0–23
**Espoo 3**	2.90 (19) / 4.10 (20)	0.0 / 3.0	0–20 / 0–14	14.67 (15) / 8.89 (18)	6.0 / 6.0	0–85 / 0–40
**Espoo 4**^**b**^	168.84 (19) / 67.88 (17)	34.0 / 20.0	0–1545 / 0–633	549.79 (14) / 240.77 (17)	54.0 / 31.0	0–5024 / 0–1721

n = The number of observations are presented in parenthesis after Mean values.

^a^Most urban.

^b^Least urban.

## 3 Results

The proportion of zero pollen concentrations decreased when moving from the most urban to the least urban sampling sites in both cities (Table 2 and S1 Fig). Both in Helsinki and in Espoo, average grass pollen concentrations were higher in the height of 1.5 meters compared to the height of 4 meters (mean 5.24 grains / m^3^ (SD 13.05) vs. 3.84 grains / m^3^ (SD 4.36) in Helsinki; P = 0.4542, and 75.71 (SD 450.40) vs. 37.42 grains / m^3^ (SD 197.37) in Espoo; P = 0.9070). This was detected both in the morning (mean 5.17 grains / m^3^ (SD 15.99) vs. 3.53 grains / m^3^ (SD 4.48) in Helsinki; P = 0.3300, and 40.66 grains / m^3^ (SD 186.09) vs. 17.08 grains / m^3^ (SD 71.97) in Espoo; P = 0.5395) and in the afternoon (5.33 grains / m^3^ (SD 7.51) vs. 4.29 grains / m^3^ (SD 4.18) in Helsinki; P = 0.9810, and 118.39 grains / m^3^ (SD 638.61) vs. 59.92 grains / m^3^ (SD 275.56) in Espoo; P = 0.6591). Some of these differences could be explained by chance.

In negative binomial regression analyses, grass pollen concentrations decreased when height of measurement increased (-1.40 grains / m^3^ per height rank, 95% CI: -3.01 to 0.21 in Helsinki and -38.29 grains / m^3^, -69.07 to -7.52 in Espoo). When the level of urban gradient, period of measurement time, day and temperature were included into the models, the effect of the measurement height on grass pollen concentrations diminished further (0.01 grains / m^3^, -1.45 to 1.48 in Helsinki and -0.28 grains / m^3^, -3.37 to 2.82 in Espoo). When the urban land use, period of measurement time, day and temperature were included into the models, the effect of measurement height on grass pollen concentrations diminished (-0.04 grains / m^3^, -1.54 to 1.48 in Helsinki and -0.31 grains / m^3^, -3.20 to 2.58 in Espoo), respectively (Table 3, models 1–3). When information only from the least urban sampling sites (Helsinki 4 and Espoo 4) were included into the models then the effect of the measurement height on grass pollen concentrations were 0.19 grains / m^3^, -1.70 to 2.08 in Helsinki and 1.92 grains / m^3^, -0.09 to 3.93 in Espoo, respectively (Table 3, model 4)

**Table 3:** The relationship between measurement height and/or environmental factors and grass pollen concentration in negative binomial regression model (Helsinki n = 293, Espoo n = 311).

	POLLEN in Helsinki (pollen grains / m^3^)			POLLEN in Espoo (pollen grains / m^3^)		
Predictors	Estimate (95% CI)	AIC / BIC	P value	Estimate (95% CI)	AIC / BIC	P value
**Model 1**		1480.2 /1491.3			2344.8 / 2356.1	
Height (1.5 vs. 4m)	-1.4013 (-3.0107, 0.2081)		0.0879	**-38.2944** (-69.0676, -7.5212)		**0.0147**
**Model 2**		1468.6 / 1494.3			2252.1 / 2278.3	
Height (1.5 vs. 4m)	0.0130 (-1.4517, 1.4777)		0.9861	-0.2769 (-3.3718, 2.8181)		0.8608
Urban gradient (1 to 4)	**1.1672** (0.5979, 1.7364)		**< .0001**	**25.9937** (18.7717, 33.2157)		**< .0001**
Sampling period (AM = 1, PM = 2)^a^	1.0656 (-0.3298, 2.4610)		0.1344	1.9500 (-2.3863, 6.2863)		0.3781
Day (weekday = 1, weekend = 2)	-0.8454 (-2.0979, 0.4071)		0.1859	0.4316 (-4.7232, 5.5864)		0.8697
Temperature (1 to 3)	-0.4256 (-1.7701, 0.9189)		0.5350	0.2515 (-2.4617, 2.9647)		0.8558
**Model 3**		1468.9 / 1494.7			2250.9 / 2277.1	
Height (1.5 vs. 4m)	-0.0339 (-1.5426, 1.4748)		0.9649	-0.3096 (-3.1987, 2.5796)		0.8337
Urban land use (m^2^; 1 to 4)^b^	**1.1944** (0.5159, 1.8730)		**0.0006**	**25.8695** (18.6804, 33.0586)		**< .0001**
Sampling period (AM = 1, PM = 2)^a^	1.0382 (-0.3697, 2.4461)		0.1484	-0.8112 (-8.4668, 6.8444)		0.8355
Day (weekday = 1, weekend = 2)	-0.8731 (-2.1962, 0.4501)		0.1959	-0.5686 (-3.7816, 2.6444)		0.7287
Temperature (1 to 2)	-0.1690 (-1.8532, 1.5151)		0.8441	3.3479 (-3.7112, 10.4070)		0.3526
**Model 4**^**c**^ **(n = 72)**		357.3 / 371.0			415.1 / 428.7	
Height (1.5 vs. 4m)	0.1923 (-1.6984 , 2.0830)		0.8420	1.9173 (-0.0917, 3.9263)		0.0614
Sampling period (AM = 1, PM = 2)^a^	0.8397 (-1.1891, 2.8685)		0.4172	3.9129 (-4.0452, 11.8710)		0.3352
Day (weekday = 1, weekend = 2)	0.4377 (-1.7799, 2.6553)		0.6989	0.4086 (-1.7331, 2.5503)		0.7084
Temperature (1 to 2)	0.8182 (-1.2586, 2.8950)		0.4400	5.9491 (-0.5423, 12.4405)		0.0725
**Model 5**		1460.1 / 1478.5			2207.5 / 2226.2	
Height (1.5 vs. 4m)	0.4103 (-0.9173, 1.7378)		0.5447	0.2209 (-2.5271, 2.9689)		0.8748
Urban gradient (0, 1)	**5.5114** (2.6940, 8.3287)		**0.0001**	**162.1680** (87.9021, 236.4338)		**< .0001**
Height * Urban gradient	**-3.7340** (-7.1612, -0.3067)		**0.0327**	**-90.8505** (-172.043, -9.6583)		**0.0283**
**Model 6**		1464.4 / 1482.8			2243.3 / 2262.0	
Height (1.5 vs. 4m)	1.5431 (-0.7519, 3.8381)		0.1876	**17.9079** (2.2672, 33.5486)		**0.0248**
Urban land use (m^2^; 1 to 4)^b^	**1.6776** (0.8502, 2.5051)		**< .0001**	**35.5400** (21.9617, 49.1184)		**< .0001**
Height * Urban land use	-1.0880 (-2.1940, 0.0179)		0.0538	**-18.1008** (-33.3051, -2.8965)		**0.0196**

^a^ AM = morning period, PM = afternoon period.

^b^ Surface area of urban land use in square meters within a 100-meter radius of the study site.

^c^ Model 4 includes information only from the least urban sampling sites in both cities (Helsinki 4 and Espoo 4).

The interaction terms included in the models were mostly statistically significant and their inclusion improved the fit of the models. This indicated that the effect of height on pollen concentrations differs depending on the level of urbanity (Table 3, models 5 and 6). Grass pollen concentrations were higher at the breathing height (i.e. 1.5m) in the least urban sites in both cities. Correspondingly, concentrations were higher at height of 4.0 meters in the most urban sites. The effect of urbanity on pollen concentrations at both heights was stronger in less urban Espoo (-90.85 grains / m^3^, -172.04 to -9.66 and -18.10 grains / m^3^, -33.31 to -2.90).

## 4 Discussion

### 4.1 Main findings

A variation in the exposure to grass pollen was extensively assessed at two different measurement heights and several urban and suburban environments. Grass pollen concentrations in the Helsinki metropolitan area in Finland varied mainly between low (< 10 pollen grains/m^3^) and moderate (10–30 pollen grains/m^3^) [24]. Exposure to grass pollen in the urban environments did not change much in relation to altitude. Although grass pollen concentrations were on average higher at the height of 1.5 meters (corresponding to the breathing height) when compared to the height of 4 meters, differences in pollen concentrations between these two heights were not significant. In contrast, urbanity was the most important factor determining differences in the observed pollen concentrations. The effect of height on pollen concentrations varied depending on the level of urbanity. Due to special nature of urban environments, pollen grains are probably more efficiently mixed in the air of cities. As a consequence of this, cities offer only a moderate place of refuge for allergic and/or asthmatic people.

### 4.2 Validity of results

The validity of result issues were discussed comprehensively in a previously published horizontally-oriented article [4]. Several sampling sites, separate morning and afternoon sampling periods and progression of sampling in relation to time made it possible to measure the spatial and temporal variations in pollen concentrations within cities. Although, the height difference between 1.5 and 4 meters is not so large compared to many previous height comparisons [5, 11]. Pollen monitoring of 1.5 and 4 meters strongly reflects the diversity of the range of outdoor activity (exposure) heights of people in urban environments (i.e. bridges, walk bridges, public spaces on different heights). Due to sampling technology, monitoring was conducted only during rainless weather conditions [25]. Weather conditions were quite favorable since there were only five days with partial interruptions due to rain within the monitoring period. Due to sensitivity of sampling method and mainly low numbers of pollen grains, small differences observed in the results should be interpreted with caution.

### 4.3 Synthesis with previous knowledge

Grass pollen concentrations measured in the Helsinki metropolitan area at two heights were mainly low in the different urban environments (Table 2). This is well in line with the study conducted at three different urban locations in Berlin, Germany [26]. In the German study, daily average grass pollen concentrations at the heights between 2 and 15 meters varied between 7.9 and 17.2 grains/m^3^. In line with these findings, weekly maximum total grass pollen concentrations varied between 0 and 30 pollen grains/m^3^ in five different urban locations at rooftop height in Las Vegas, US [27]. However, grass pollen concentrations can vary substantially across the urban gradient. In the least urban environments (like site 4 in Espoo) inhabitants can be exposed to grass pollen concentrations high enough to elicit reactions among the majority of allergic and/or asthmatic people. Similarly, Werchan et al. [28] observed substantial total differences (up to 306%) in grass pollen sedimentations on the collection surfaces between different urban locations in Berlin, Germany.

Mean grass pollen concentrations were generally higher in the height of 1.5 meters compared to the height of 4 meters, both in Helsinki and in Espoo (Table 2), although not always statistically significant. This is of importance as the breathing height is around the 1.5 meters, while the stationary monitoring stations are usually located on roofs of large buildings. In particular, rurality (i.e. variation in the amount local vegetation) increased significantly the pollen concentrations at the height of 1.5 meters in both cities (Table 3). There were no statistically significant differences in the daily average grass pollen counts between the two samplers (ground level vs. 16 m) in Badajoz, Spain [11]. The roof /ground ratio was 0.99. No significant difference was observed either between the total grass pollen data obtained from two samplers placed at the height of 1.5 and 15 m in Alassio, Italy [12]. In contrast, the sampler placed at 5 m captured significantly less total grass pollen than samplers placed at 1.5 m [12]. In addition, a study conducted using two samplers placed at two different heights (1.5 and 15 m) in Córdoba, Spain [6] showed that there were significant differences in the grass concentrations between heights, the mean annual values at 1.5 m being generally higher. Similarly, mean annual grass pollen counts were 4.4 times higher at ground level compared to roof level (15 m) in Turku, Finland [5]. Recently, Rojo et al. [13] showed that pollen concentrations decreased with altitude. Reduction and fluctuation in pollen volume was most pronounced up to height of ten meters and fluctuations stabilized soon above that.

Generally, mean pollen concentrations in the most urban sites (1–3) in both cities were higher at the height of 4 meters compared to the height of 1.5 meters in the mornings (Table 2). These observations were verified by the results from the models using interaction terms. Urbanity increased pollen concentrations significantly at the height of 4.0 meters in both cities (Table 3). This can be partly explained by upward-going air currents during warm afternoon and evening periods and/or sporadic turbulences caused by special characteristics of urban environments, simultaneous rise of pollen concentrations in the upper layers of the atmosphere and relatively slow fall of pollen grains during night and morning periods [6]. A study conducted in Aarhus, Denmark, and London, UK showed that median street level (1.5 m) grass pollen concentrations in both cities were lower than roof-level (11 and 18 m) concentrations [9]. Median grass pollen street/roof ratios based on one sampling site were 0.89 in Aarhus and 0.77 in London, respectively. Unlike in this study, street level concentrations were lower than roof-level concentrations in Aarhus and London between mid-day and late evening.

In the present study, mean pollen concentrations were highest at the height of 1.5 meters in the afternoon (Table 2). Mean pollen concentrations increased from the morning to the afternoon periods in both cities, reaching its peak values on the least urban sampling sites during afternoon period. This is partly in line with previous study from Turku, Finland, where grass pollen had a second peak at 16:00–18:00 hours [29], although, the highest peak occurred between 8:00 and 12:00 hours at both heights (ground vs. 15 m). In contrast, Simoleit et al. [30] observed that the highest peak of grass pollen was on midday or in the afternoon at height of 5 meter in Berlin, Germany.

Due to the characteristics of sampling site, weather conditions, time and season the distribution and abundance of grass pollen grains can vary substantially between sampling sites regardless of the sampling height [12, 31]. However, it seems that grass pollen grains are reasonably well mixed vertically especially in the most urban environments where pollen concentrations are at the lowest. It is assumed that the vertical gradient is not so prominent in environments, where local sources of pollen are lacking and the exposure to pollen is mainly based on better vertically mixed pollen coming from a longer distance (i.e. from environments outside the immediate neighborhood of sampling sites) and gradually settling down due to calm weather conditions [5]. Instead of altitude, urbanity was the strongest determinant of grass pollen grains. In a negative binomial regression analyses, grass pollen concentrations decreased when altitude increased. When the level of urban gradient or urban land use, period of time, day and temperature were included into the models, the effect of height on grass pollen concentrations diminished considerably. Due to difference in mean pollen levels and trends between the most urban sampling sites (sites 1–3) and the least urban sampling site (sites 4), we analyzed data using the information from the least urban sampling sites (Table 3, model 4) in order to elucidate whether the effect of altitude becomes visible in the environment of lower urbanization. The results suggest that pollen counts would appear to increase with increasing altitude. This observation, together with varying circumstances and pollen concentrations, underlines the importance of more detailed data collection and analyses.

According to interaction models, the effect of height was different depending on the level of urbanity. This strongly refers to the crucial role of urbanization in the determination of the distribution and abundance of grass pollen grains in cities. The results of this study are in line with the previous horizontally-oriented study conducted in Helsinki Metropolitan Area [4]. Differences in growth conditions can lead to dissimilarities in flowering outcomes between urban and suburban/rural environments. Previously, Rodríguez-Rajo et al. [32] observed based on monitoring conducted at roof level that pollen season started earlier and lasted longer but pollen peaks and annual pollen index were generally lower in urban compared to less urban sampling site in Poznan, Poland.

## 5 Conclusions

Grass pollen concentration varied mainly between low and moderate levels, and the concentrations did not change considerably in relation to altitude across urban space, although, height may affect the abundance and distribution of grass pollen in the urban environments. Other factors like urban gradient and urban land use, can have a stronger impact on grass pollen concentrations in the urban environments. Urbanity seems to modify the effects of height on pollen concentrations. More studies on the determinants, abundance and distribution of pollen grains in the different environments are needed.

## Supporting information

S1 Fig (PDF)

S1 Table Progress figures of pollen monitoring in Helsinki and Espoo. X illustrates the starting time of sampling in the sampling sites.
(DOCX)
