# Supplementary material for: The effect of sampling height on grass pollen concentrations in different urban environments in the Helsinki Metropolitan Area, Finland
Source: PLoS One. 2020 Sep 29;15(9):e0239726. doi: 10.1371/journal.pone.0239726 (PMC7523945; doi:10.1371/journal.pone.0239726)
Supplement: S1 Fig — (PDF) [file pone.0239726.s001.pdf]

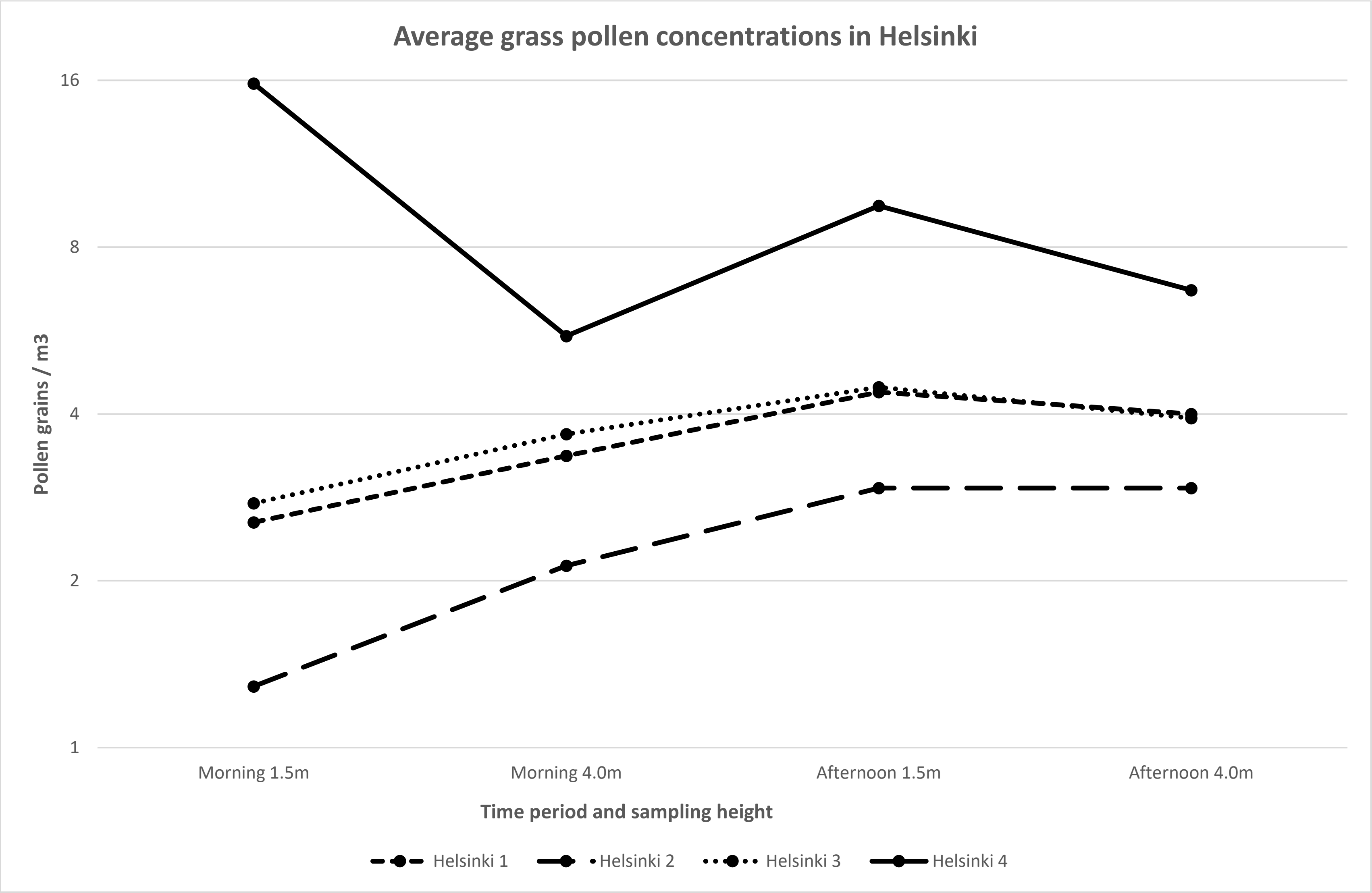
Average grass pollen concentrations in Helsinki
16
8
4
2
1
Pollen grains / m3
Morning 1.5m
Morning 4.0m
Afternoon 1.5m
Afternoon 4.0m
Time period and sampling height
Helsinki 1
Helsinki 2
Helsinki 3
Helsinki 4

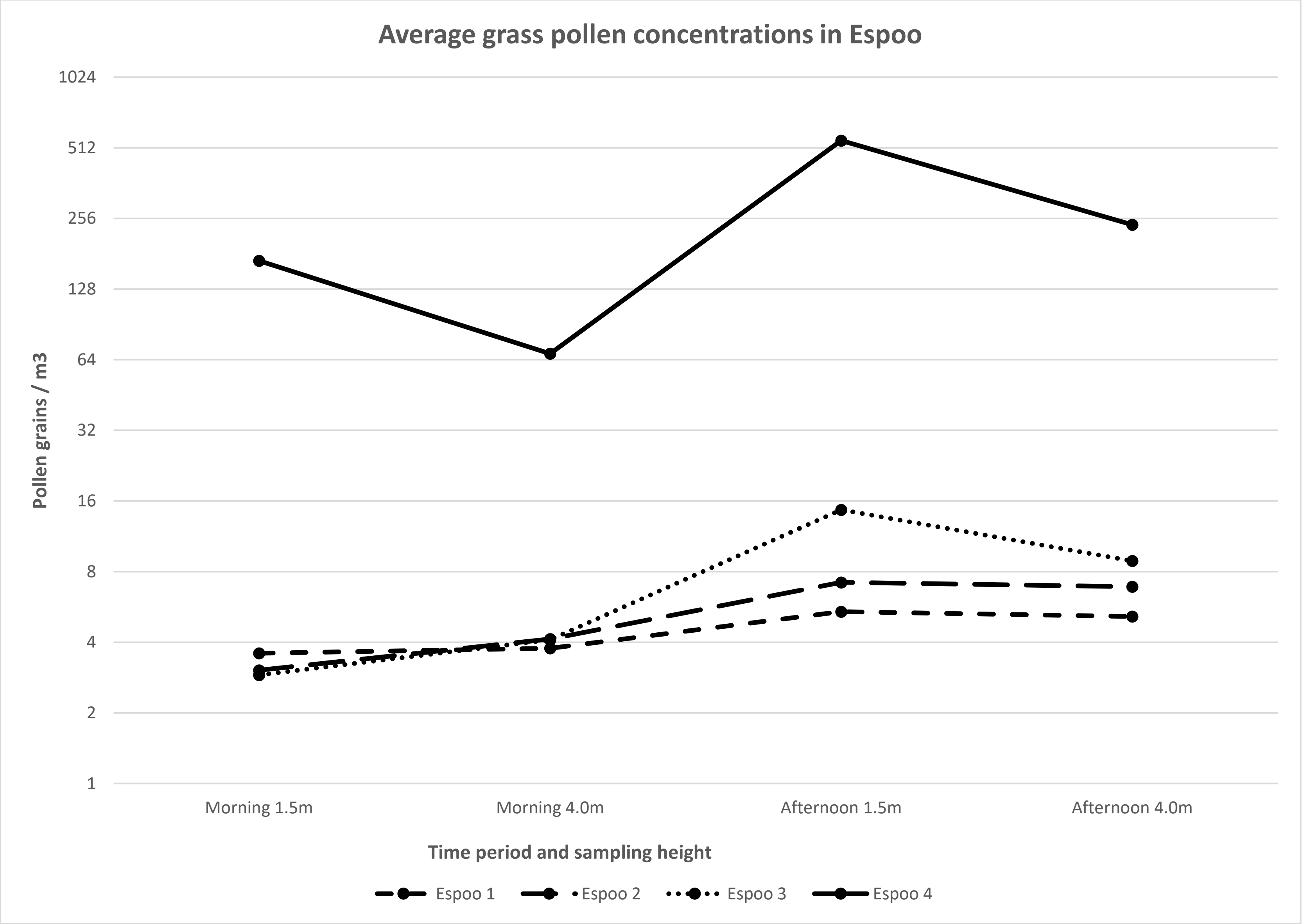
Average grass pollen concentrations in Espoo
1024
512
256
128
64
32
16
8
4
2
1
Pollen grains / m3
Morning 1.5m
Morning 4.0m
Afternoon 1.5m
Afternoon 4.0m
Time period and sampling height
Espoo 1
Espoo 2
Espoo 3
Espoo 4
