# Supplementary material for: The effect of sampling height on grass pollen concentrations in different urban environments in the Helsinki Metropolitan Area, Finland
Source: PLoS One. 2020 Sep 29;15(9):e0239726. doi: 10.1371/journal.pone.0239726 (PMC7523945; doi:10.1371/journal.pone.0239726)
Supplement: S1 Table — X illustrates the starting time of sampling in the sampling sites. (DOCX) [file pone.0239726.s002.docx]

**S1 Table. Progress figures of pollen monitoring in Helsinki and Espoo. X illustrates the starting time of sampling in the sampling sites.**

| **Time** | **Sampling site 1** | **Sampling site 2** | **Sampling site 3** | **Sampling site 4** |
| --- | --- | --- | --- | --- |
| Helsinki 1^st^ day (morning) |  |  |  |  |
| 8.00 am | x |  |  | x |
| 9.00 am |  |  | x |  |
| 10.00 am |  | x |  |  |
| 11.00 am |  |  |  |  |
| Helsinki 1^st^ day (afternoon) |  |  |  |  |
| 1.00 pm | x |  |  | x |
| 2.00 pm |  |  | x |  |
| 3.00 pm |  | x |  |  |
| 4.00 pm |  |  |  |  |
| Helsinki 2^nd^ day (morning) |  |  |  |  |
| 11.00 am | x |  |  | x |
| 8.00 am |  |  | x |  |
| 9.00 am |  | x |  |  |
| 10.00 am |  |  |  |  |
| Helsinki 2^nd^ day (afternoon) |  |  |  |  |
| 4.00 pm | x |  |  | x |
| 1.00 pm |  |  | x |  |
| 2.00 pm |  | x |  |  |
| 3.00 pm |  |  |  |  |
| Helsinki 3^rd^ day (morning) |  |  |  |  |
| 10.00 am | x |  |  | x |
| 11.00 am |  |  | x |  |
| 8.00 am |  | x |  |  |
| 9.00 am |  |  |  |  |
| Helsinki 3^rd^ day (afternoon) |  |  |  |  |
| 3.00 pm | x |  |  | x |
| 4.00 pm |  |  | x |  |
| 1.00 pm |  | x |  |  |
| 2.00 pm |  |  |  |  |
| Helsinki 4^th^ day (morning) |  |  |  |  |
| 9.00 am | x |  |  | x |
| 10.00 am |  |  | x |  |
| 11.00 am |  | x |  |  |
| 8.00 am |  |  |  |  |
| Helsinki 4^th^ day (afternoon) |  |  |  |  |
| 2.00 pm | x |  |  | x |
| 3.00 pm |  |  | x |  |
| 4.00 pm |  | x |  |  |
| 1.00 pm |  |  |  |  |
| Espoo 1^st^ day (morning) |  |  |  |  |
| 8.00 am |  |  |  |  |
| 9.00 am | x | x |  |  |
| 10.00 am |  |  | x |  |
| 11.00 am |  |  |  | x |
| Espoo 1^st^ day (afternoon) |  |  |  |  |
| 1.00 pm |  |  |  |  |
| 2.00 pm | x | x |  |  |
| 3.00 pm |  |  | x |  |
| 4.00 pm |  |  |  | x |
| Espoo 2^nd^ day (morning) |  |  |  |  |
| 11.00 am |  |  |  |  |
| 8.00 am | x | x |  |  |
| 9.00 am |  |  | x |  |
| 10.00 am |  |  |  | x |
| Espoo 2^nd^ day (afternoon) |  |  |  |  |
| 4.00 pm |  |  |  |  |
| 1.00 pm | x | x |  |  |
| 2.00 pm |  |  | x |  |
| 3.00 pm |  |  |  | x |
| Espoo 3^rd^ day (morning) |  |  |  |  |
| 10.00 am |  |  |  |  |
| 11.00 am | x | x |  |  |
| 8.00 am |  |  | x |  |
| 9.00 am |  |  |  | x |
| Espoo 3^rd^ day (afternoon) |  |  |  |  |
| 3.00 pm |  |  |  |  |
| 4.00 pm | x | x |  |  |
| 1.00 pm |  |  | x |  |
| 2.00 pm |  |  |  | x |
| Espoo 4^th^ day (morning) |  |  |  |  |
| 9.00 am |  |  |  |  |
| 10.00 am | x | x |  |  |
| 11.00 am |  |  | x |  |
| 8.00 am |  |  |  | x |
| Espoo 4^th^ day (afternoon) |  |  |  |  |
| 2.00 pm |  |  |  |  |
| 3.00 pm | x | x |  |  |
| 4.00 pm |  |  | x |  |
| 1.00 pm |  |  |  | x |
